# TSANN-TG: Temporal–Spatial Attention Neural Networks with Task-Specific Graph for EEG Emotion Recognition

**DOI:** 10.3390/brainsci14050516

**Published:** 2024-05-20

**Authors:** Chao Jiang, Yingying Dai, Yunheng Ding, Xi Chen, Yingjie Li, Yingying Tang

**Affiliations:** 1 School of Communication and Information Engineering, Shanghai University, Shanghai 200444, China; jiangchao@shiep.edu.cn (C.J.); chenx07_shu@shu.edu.cn (X.C.); 2 College of Electronics and Information Engineering, Shanghai University of Electric Power, Shanghai 200090, China; dai_yy@mail.shiep.edu.cn (Y.D.); dingyh0526@mail.shiep.edu.cn (Y.D.); 3 College of International Education, Shanghai University, Shanghai 200444, China; 4 School of Life Sciences, Shanghai University, Shanghai 200444, China; 5 Institute of Biomedical Engineering, Shanghai University, Shanghai 200444, China; 6 Shanghai Mental Health Center, Shanghai 200030, China; yytang0522@gmail.com

**Keywords:** EEG, emotion recognition, graph convolutional neural network, temporal–spatial attention, task-specific graph

## Abstract

Electroencephalography (EEG)-based emotion recognition is increasingly pivotal in the realm of affective brain–computer interfaces. In this paper, we propose TSANN-TG (temporal–spatial attention neural network with a task-specific graph), a novel neural network architecture tailored for enhancing feature extraction and effectively integrating temporal–spatial features. TSANN-TG comprises three primary components: a node-feature-encoding-and-adjacency-matrices-construction block, a graph-aggregation block, and a graph-feature-fusion-and-classification block. Leveraging the distinct temporal scales of features from EEG signals, TSANN-TG incorporates attention mechanisms for efficient feature extraction. By constructing task-specific adjacency matrices, the graph convolutional network with an attention mechanism captures the dynamic changes in dependency information between EEG channels. Additionally, TSANN-TG emphasizes feature integration at multiple levels, leading to improved performance in emotion-recognition tasks. Our proposed TSANN-TG is applied to both our FTEHD dataset and the publicly available DEAP dataset. Comparative experiments and ablation studies highlight the excellent recognition results achieved. Compared to the baseline algorithms, TSANN-TG demonstrates significant enhancements in accuracy and F1 score on the two benchmark datasets for four types of cognitive tasks. These results underscore the significant potential of the TSANN-TG method to advance EEG-based emotion recognition.

## 1. Introduction

Emotions, arising from both conscious and unconscious perceptions of stimuli, profoundly impact our mental states. Positive emotions serve as catalysts for enhancing our daily productivity, while negative emotions can sway decision-making, focus, and overall well-being. The study of emotion recognition stands at the forefront of interdisciplinary research, drawing significant attention due to its diverse applications across psychology, physiology, neuroscience, and computer science [1]. A brain–computer interface (BCI) enables a user to communicate directly with a computer using only the central nervous system. An affective BCI (aBCI) monitors and/or regulates the emotional state of the brain. In a closed-loop aBCI system, it includes signal acquisition, signal processing, feature extraction, emotion recognition, and brain stimulation [2]. During aBCI, emotions can be represented using categories, e.g., Ekman’s six basic emotions (anger, disgust, fear, happiness, sadness, and surprise), or continuously in the 2D space of arousal and pleasure (or valence), or the 3D space of arousal, pleasure (or valence), and dominance. Most aBCI experiments were performed in controlled laboratory environments, using deliberately designed settings to elicit specific emotions of the subjects, while simultaneously recording their brain and other physiological signals for analysis using superior machine/deep learning models.

Electroencephalography (EEG) has emerged as a pivotal tool in unraveling the cognitive and affective processes underlying emotional experiences. With its noninvasive nature, relatively low cost, and high temporal resolution, EEG offers invaluable insights into neural systems, rehabilitation engineering, and affective computing, illuminating how emotions are processed and manifested in the brain [3,4]. The landscape of EEG-based emotion recognition is rapidly evolving, with a concerted effort to deepen our understanding of the neural substrates of human emotions. Current endeavors are centered around harnessing advanced algorithms and machine learning techniques to distill meaningful insights from EEG data. This enables the precise identification of emotional states and their cognitive correlates, facilitating a nuanced comprehension of human affective experiences [5,6].

In EEG-based emotion recognition research, fundamental methodologies encompass feature extraction, classification, or regression analysis. Numerous studies emphasize the profound impact of EEG signal characteristics across temporal and spatial domains on pattern recognition efficacy [7,8,9,10]. For instance, Zheng et al. highlight the enhancement of emotion classification accuracy through leveraging specific EEG features, emphasizing the pivotal role of critical frequency bands and channels in advancing our understanding of emotional processing in the brain [11,12]. Furthermore, research by Schirrmeister et al. explores the potential of deep learning and convolutional neural networks (CNNs) in decoding and visualizing EEG data. They demonstrate the superiority of CNNs in handling spatiotemporal relationships, effectively capturing features within EEG signals, and improving decoding accuracy [13,14]. This holistic approach addresses the limitations of conventional methods that often focus solely on either the temporal or spatial aspects of EEG signals, thus enhancing the accuracy and robustness of emotion recognition models.

In recent years, graph neural networks (GNNs) have emerged as a prominent tool in deep learning, driving significant advancements across various domains. In the realm of EEG-based emotion recognition, GNNs offer a unique advantage by simultaneously considering the local neighborhoods of nodes and the global structure of the entire graph [15,16,17,18]. This enables better capture of both local and global information embedded in EEG data, thereby enhancing the model’s understanding and representation of EEG data and providing a promising avenue for analyzing complex brain networks and extracting nuanced emotional features [19,20,21,22]. Several studies have illustrated the effectiveness of GNNs in boosting emotion recognition accuracy and elucidating the underlying neural mechanisms. 

Despite the satisfactory performance demonstrated by both CNN-based and GNN-based network models in EEG emotion recognition, there is still a pressing need to further enhance the effectiveness of extracting and integrating temporal–spatial features. Continued research efforts in this direction are crucial for advancing the state-of-the-art in EEG emotion recognition [23,24,25].

In this paper, we propose a novel neural network architecture named TSANN-TG (temporal–spatial attention neural network with a task-specific graph). This architecture is specifically designed to enhance the feature extraction process and integrate temporal–spatial features more effectively. By amalgamating the temporal and spatial information encoded in EEG signals with the potent feature-learning capabilities of CNNs or GNNs, it can capture the intricate spatiotemporal relationships present in EEG data, achieving more robust and interpretable emotion recognition systems. Specifically, TSANN-TG leverages the distinct temporal scales of features from each electrode in EEG signals, supplemented by the attention mechanisms, to achieve efficient feature extraction. By constructing task-specific adjacency matrices, the network captures dynamic changes in dependency information between EEG channels across different emotional states, allowing for the extraction of targeted temporal–spatial features. Moreover, TSANN-TG emphasizes the integration of features at multiple levels with a specific focus, ultimately leading to improved performance in emotion recognition tasks.

This paper follows the subsequent structure: Section 2 introduces CNNs for EEG learning, graph learning and GNNs for emotion recognition; in Section 3, the EEG methods utilized in our study are elucidated; Section 4 provides a comprehensive overview of the materials used and outlines the experiments conducted; the results of these experiments are presented and analyzed in Section 5; Section 6 offers a discussion on the findings; and lastly, Section 7 concludes our research.

## 2. Related Work

### 2.1. CNNs for EEG Learning

With the rapid development of deep learning technologies, CNNs have achieved remarkable success in various fields, such as computer vision and natural language processing. In recent years, researchers have begun to apply these techniques to the learning and analysis of EEG signals, exploring their potential value in neuroscience and neurology. The application of CNNs to EEG signal processing presents distinct challenges. Unlike static images, EEG signals exhibit dynamic temporal characteristics and a relatively low signal-to-noise ratio. By modifying CNN architectures to accommodate these temporal dynamics, meaningful features can be extracted from EEG signals acquired through scalp electrode measurements. This adaptation is crucial for effectively capturing the temporal sequences inherent in EEG signals, which are essential for decoding task-related information and emotion recognition.

CNNs have significant advantages in feature extraction from EEG signals. Through the combination of convolutional layers and pooling layers, CNNs can automatically learn features on different frequencies and time scales to extract information relevant to a specific task. For example, in emotion recognition tasks, CNNs can effectively capture features reflecting emotional states in EEG signals, providing strong support for emotion classification. CNNs also showed good performance in EEG signal classification and recognition. By connecting the full connection layer and softmax function after the convolutional layer, CNNs can map the learned features to specific classification labels, thus achieving automatic classification of EEG signals. This end-to-end learning method enables CNNs to learn high-level feature representations directly from raw data in EEG signal analysis, avoiding the complicated process of manually designing features and improving the accuracy and efficiency of classification.

Bashivan et al. pioneered the transformation of EEG activities into topology-preserving multi-spectral images, which were subsequently fed into CNNs for classification research [23]. This innovative approach, inspired by image processing, outperformed traditional models like support vector machine (SVM) and deep belief network (DBN) in the context of mental load classification tasks. The proposed approach is designed to preserve the spatial, spectral, and temporal structure of EEG, which leads to finding features that are less sensitive to variations and distortions within each dimension. Empirical evaluation of the cognitive load classification task demonstrated significant improvements in classification accuracy over current state-of-the-art approaches in this field.

Schirrmeister et al. contributed a comprehensive discussion on using deep CNNs for end-to-end EEG analysis [13,14]. They not only addressed the design and training of CNNs for EEG decoding but also emphasized the visualization of informative EEG features. Their findings demonstrated that deep CNNs trained end-to-end achieved comparable performance to algorithms utilizing hand-engineered features for decoding task-specific information. And it also demonstrated that CNNs indeed learned to use spectral power modulations in the alpha, beta, and high gamma frequencies. It proved useful for spatially mapping the learned features by revealing the topography of the causal contributions of features in different frequency bands to the decoding decision. Moreover, the Deep ConvNet and Shallow ConvNet architectures they proposed have served as foundational models for subsequent research in EEG analysis. 

Lawhern et al. introduced EEGNet, a compact CNN designed specifically for EEG-based brain–computer interfaces [26]. This innovative model integrates depth-wise and separable convolutions, leveraging well-established EEG feature extraction concepts like optimal spatial filtering and filter-bank construction. Their approach demonstrated significant effectiveness across diverse experiments, showcasing the potential of integrating computer vision techniques into EEG analysis. By considering the distinctive characteristics of EEG signals, EEGNet facilitates the extraction of representative features from raw EEG data. This optimized feature extraction captures vital information pertaining to different cognitive states or emotions encoded in EEG signals, thereby providing richer and more effective inputs for subsequent classification tasks. 

Ding et al. proposed TSception, a multi-scale CNN architecture specifically tailored to capture the temporal dynamics and spatial asymmetry inherent in EEG data for emotion recognition [27,28]. By incorporating temporal dynamics and spatial asymmetry into the feature extraction process, they achieved a more comprehensive representation of EEG data. TSception demonstrated promising performance on DEAP (a dataset for emotion analysis using EEG, physiological, and video signals), with trial-wise 10-fold cross-validation, achieving 61.57% accuracy and a 63.24% F1 score on arousal, and 59.14% accuracy and a 62.33% F1 score on valence. Through extensive comparisons with various methods, their proposed approach showed a high degree of generality in the arousal–valence prediction task. 

Although CNNs have traditionally been associated with computer vision tasks, their adaptation to EEG analysis presents exciting opportunities for unraveling new insights into brain dynamics. With careful modification and evaluation, CNNs are poised to serve as a robust framework for decoding EEG signals and advancing our understanding of neural processes.

### 2.2. Graph Learning and GNNs for EEG Emotion Recognition

Graph learning is a method of learning nodes or representations of graphs from graph structure data. Node features are vectors that describe the attributes of each node in a graph. The adjacency matrix describes the connectivity between nodes in a graph. Aggregation functions are used to combine the feature information of a node with that of its neighboring nodes to generate a new node representation.

Formally, a graph is represented as $G=\left(V,E\right)$, where $V=\left\{v_{1},...,v_{N}\right\}\in R^{N\times d}$, representing a node feature matrix, is a set of $N=\left|V\right|$ nodes, and E∈V×V is a set of $M=\left|E\right|$ edges between nodes. Here, $v_{k}\in R^{d}$ corresponds to the feature vector of a node $v_{k}$, providing additional information beyond the graph’s connectivity structure. An edge from node u∈V to node v∈V is typically represented as $\left(u,v\right)\in E$. The adjacency matrix, denoted as $A\in R^{N\times N}$, is employed to capture the graph’s connectivity structure, with its elements $A\left(i,j\right)$ representing the presence (or weight) of an edge between nodes i and j. Graphs can exhibit various characteristics, including directionality and the weighting of edges, giving rise to directed/undirected and weighted/unweighted graphs.

Scarselli et al. offer a comprehensive overview of the graph neural network (GNN) model, elucidating its architecture, principles, and applications [29]. By delving into the propagation of information within graph structures, the authors establish a theoretical framework that underpins GNN operations. This foundational comprehension is crucial for researchers and practitioners aiming to utilize GNNs across diverse domains.

Bruna et al. provide an in-depth exploration of two distinct methodologies for processing graph-structured data: spectral networks and locally connected networks [30]. Spectral networks utilize the spectral domain to conduct convolution-like operations on graph data, extending traditional CNN concepts to graphs. This framework not only facilitates efficient feature extraction but also enables effective information propagation across graph nodes. In contrast, locally connected networks operate from a spatial perspective, applying convolutional operations directly to local neighborhoods within the graph. This spatial approach offers insights into the spatial relationships between nodes and enables more localized feature extraction. By examining and comparing these methodologies, the study contributes to our understanding of GNNs and offers insights for developing more effective and efficient models for graph-based learning tasks.

Defferrard et al. introduce a significant advancement in graph-based deep learning by proposing a novel approach for directly applying CNNs to graph-structured data, termed fast localized spectral filtering [31]. This method leverages spectral graph theory to define localized convolution operations on graphs, enabling efficient and scalable convolution operations, thus extending the capabilities of CNNs to non-Euclidean domains. By decomposing the convolutional kernel into localized spectral filters, this method achieves computational efficiency and scalability, rendering it feasible to apply CNNs to large-scale graph data with millions of nodes and edges.

Kipf et al. introduce a pivotal method for semi-supervised learning on graph-structured data using graph convolutional networks (GCNs) [32]. It introduces a scalable and efficient training algorithm for GCNs, which leverages the graph Laplacian matrix to define graph convolution operations and optimize model parameters.

Song et al. proposed dynamical graph convolutional neural networks (DGCNNs) to address challenges in modeling the temporal dynamics and spatial dependencies inherent in EEG signals for emotion recognition [33]. By leveraging graph convolutional neural networks dynamically, they effectively captured the temporal evolution of EEG signals and the interrelatedness between EEG electrodes. Extensive experiments conducted on the SJTU emotion EEG dataset (SEED) and DREAMER dataset demonstrated superior performance compared to state-of-the-art methods. On the SEED database, the proposed method achieved an average recognition accuracy of 90.4% for subject-dependent experiments and 79.95% for subject-independent cross-validation. Similarly, on the DREAMER database, average accuracies of 86.23%, 84.54%, and 85.02% were, respectively, obtained for valence, arousal, and dominance classifications.

Zhang et al. proposed a fusion strategy grounded in graph theory, which integrates features from multiple frequency bands into a unified representation for emotion recognition. This fusion approach enables the amalgamation of complementary information across different frequency bands, resulting in enhanced performance in emotion classification [34]. Tian et al., on the other hand, utilized various functional connectivity features derived from EEG data, such as coherence and phase synchronization, to augment the discriminative power of the graph convolutional neural network (GCN) model [35]. While both studies employed segment-wise data partitioning, which often yields superior recognition results, their GNN-based methods showed marked improvement in effectiveness. Additionally, Zhang et al. introduced a robust framework for extracting EEG frequency band features and integrating a self-adaptive graph construction [36]. This framework adeptly captures intricate relationships between EEG channels, encoding them into a graph structure, thereby boosting the generalization capability of the emotion recognition system. Compared to alternative methods, these approaches demonstrated noteworthy performance enhancements.

Graph learning demonstrates powerful capabilities with broad implications for applications in domains such as social network analysis, recommendation systems, and bioinformatics [37,38,39]. The EEG data collection process essentially maps brain activity onto a graph-like structure, where each node corresponds to an electrode location on the scalp, and edges represent the interactions and connectivity between different electrodes. By leveraging graph learning techniques adeptly, we can exploit the inherent characteristics of this graph structure to gain a more comprehensive understanding of brain activity patterns across various emotional states, thereby driving advancements in both research and practical applications within the field of affective computing.

## 3. Methods

**Figure 1:**
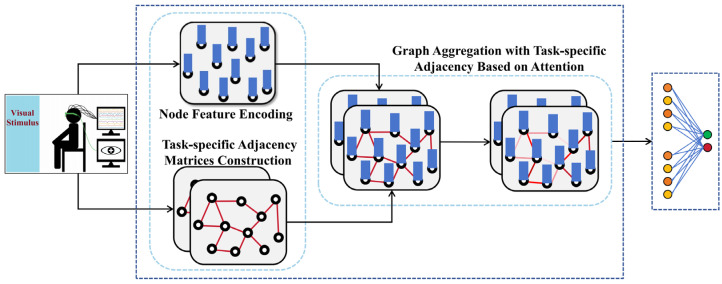
Overall architecture of TSANN-TG.

In this section, we provide a comprehensive overview of our proposed TSANN-TG framework. The overall architecture of TSANN-TG is depicted in Figure 1. TSANN-TG consists of three primary functional components: the node-feature-encoding-and-task-specific-adjacency-matrices-construction block, the graph-aggregation block, and the graph-feature-fusion-and-classification block.

**Figure 2:**
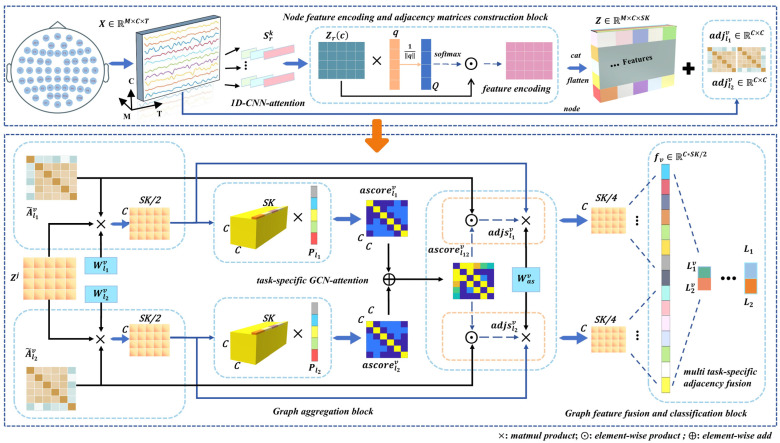
Detailed architecture of TSANN-TG. The multi-scale 1D CNNs with attention are designed to capture frequency-specific information for feature extraction and subsequently construct node feature representations for the graph network. Depending on the specific task requirements, different functional connectivity matrices are utilized to build task-specific adjacency matrix groups, enabling the exploration of various connection scenarios. Next, the task-specific adjacency matrices and node features pass through the task-specific GCN–attention component, resulting in a set of task-specific features. Finally, the features obtained from different adjacency matrix groups are effectively merged, allowing us to utilize the network for classification tasks. A detailed explanation of each block is provided in Section 3.

Illustrated in Figure 2, we detail the architecture of TSANN-TG. The node-feature-encoding-and-task-specific-adjacency-matrices-construction block focuses on extracting features from EEG data and building adjacency matrices. It employs 1D CNNs (one-dimensional convolutional neural networks) in conjunction with an attention mechanism to capture pertinent temporal–spatial information from EEG signals [40,41,42,43]. Furthermore, it utilizes various emotional stimuli to construct adjacency matrices from EEG signals, aiming to effectively encode EEG features and represent the relationships between them.

The graph-aggregation block is dedicated to enhancing and aggregating features from neighboring nodes. It employs GCNs with an attention mechanism to incorporate task-specific information into the graph [44,45,46,47]. By doing so, discriminative features are amplified and effectively integrated. This step is crucial for capturing complex relationships and dependencies among nodes in the graph.

In this final block, the fused features from different graph representations are combined and utilized for classification tasks. This achieves feature fusion across various graph representations and executes the final classification. This step ensures that the model effectively integrates information from different representations to make accurate predictions.

The TSANN-TG framework is designed to effectively process EEG data for emotion recognition tasks. It starts with feature extraction and adjacency matrices construction, followed by feature aggregation and enhancement through GCNs with attention, and concludes with feature fusion and classification to produce the final predictions. This comprehensive approach aims to leverage both temporal–spatial information from EEG signals and the structural relationships between nodes in the graph for accurate emotion recognition.

### 3.1. Node Feature Encoding and Adjacency Matrices Construction

Indeed, the cornerstone of GCN lies in its adept utilization of both node features and adjacency matrices. Analogously, we conceptualize EEG signals as a graph structure, wherein each electrode channel serves as a node, and the features extracted from each channel represent the attributes of its corresponding node. In this subsection, we present two significantly more effective components, namely node feature encoding based on 1D CNNs attention, which provides a more efficient means of encoding node characteristics, and task-specific adjacency matrices construction, which involves constructing adjacency matrices based on EEG signals elicited by different cognitive stimuli. 

#### 3.1.1. Node Feature Encoding Based on 1D CNNs Attention

We proceed under the assumption that we have been provided with EEG datasets $D^{i}=\left\{\left(X^{1},y^{1}\right),\ldots ,\left(X^{M_{i}},y^{M_{i}}\right)\right\}$, where M denotes the total number of recorded trials for subject *i*. The input matrix $X^{j}\in R^{C\times T}$ of trial j, $1\leq j\leq M_{i}$ contains the preprocessed signals of *C*-recorded electrodes and *T*-discretized time steps recorded per trial. The corresponding class label of trial j is denoted by $y^{j}$. It takes values from a set of L class labels, in our case, corresponding to the stimuli in each trial, for example, $\forall y^{j}:y^{j}\in L=\left\{l_{1}=’positive’,l_{2}=’negative’\}\right.$.

Our node feature encoding component employs parallel multi-scale 1D temporal convolutional filters with *K*-kernels, each filter length chosen as a ratio coefficient r of the data’s sampling rate $f_{s}$. Here, we use a total of three scales $\left(r\in \left[1,2,3\right]\right)$, with nine-level kernels $\left(k=\left[1,...,9\right]\right)$ for each scale. The size of the *k*-th kernels in the *r*-th level scale is denoted as

(1) $$S_{r}^{k}={\left(0.5\right)}^{r}\cdot f_{s}.$$

Following the application of the 1D CNNs layer, we incorporate a novel technique, which involves taking the logarithm of the average pooled square of the representations to effectively capture power features [14,28]. The 1D CNNs filters function as advanced digital filters, proficiently generating filtered signals across various frequency bands [26]. After squaring these filtered signals, we meticulously calculate their corresponding power values. Subsequently, a judicious application of the logarithmic activation function optimizes the representation of power characteristics within the EEG signals. 

**Figure 3:**
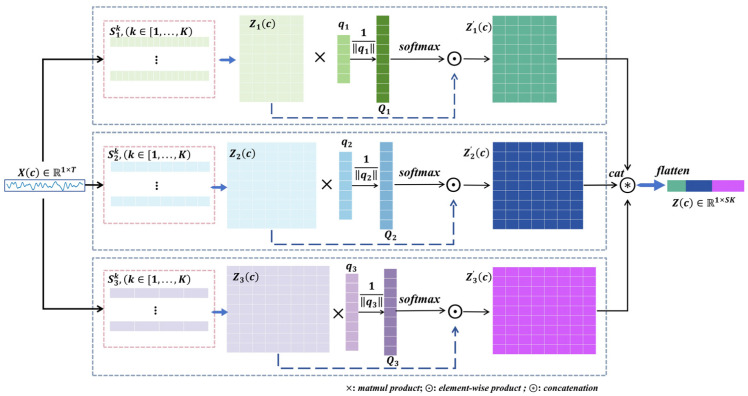
Node feature encoding based on 1D CNNs attention. $X\left(c\right)\in R^{1\times T}$ is the EEG signal of one node/channel/electrode. ×,  ⨀ and ⊛ denote matrix multiplication, element-wise product and concatenation, respectively. We considered a series of filters of three-level scales and nine-level kernels. In the attention score stage, q is a trainable projection vector. By matrix multiplication and $softmax\left(\cdot \right)$, we obtain Q, which is the attention score estimating the scalar projection values of each kernel to the projection vector. We used ⨀ to update the $Z_{r}\left(c\right)\in R^{K\times F_{k}}$ and then used $cat\left(\cdot \right)$ and $flatten\left(\cdot \right)$ to reshape features.

Let $X^{j}\left(c\right)\in R^{1\times T}$ represent the time steps for the *c*-th node of the *j*-th trial of EEG signals. For a certain 1D CNNs layer of the *r*-th level scale, let $Z_{r}\left(c,k\right)\in R^{1\times F_{k}^{r}}$ denote the output of the *k*-th level kernel, where $F_{k}^{r}$ is the length of the feature. $Z_{r}\left(c,k\right)$ is defined as

(2) $$Z_{r}\left(c,k\right)=F\left(X^{j}\left(c\right);\theta \right)=\Psi_{logS}(\Phi_{1DCNN}(X^{j}\left(c\right),\;S_{k}^{r})),$$

where $F\left(\cdot \right)$ represents the mathematical operation and θ signifies the parameter of the function. Specifically, $\Phi_{1DCNN}$ denotes the 1D CNNs function, and $\Psi_{logS}$ represents the square and logarithmic function. Then, we can get $Z_{r}\left(c\right)\in R^{K\times F^{r}}$, as illustrated in Figure 3. For the *r*-th level scale, $z_{r}^{k}$ is the feature matrix with row vectors $z_{r}^{1},{\;z}_{r}^{2}$, $...,z_{r}^{9}$, each of which corresponds to the *k*-th kernel filter. We first compute the normalization scalar projection of $z_{r}^{k}$ on $q_{r}\in R^{F^{r}}$, where $q_{r}\;$ is a trainable projection vector, resulting in the attention score $Q_{r}={\left[Q_{r}^{1},Q_{r}^{2},...,Q_{r}^{9}\;\right]}^{T}$ with each $Q_{r}^{k}$ measuring the scalar projection value of each kernel on the projection vector $q_{r}$

(3) Qr=Zrcqrqr,

where $\left\|q_{r}\right\|$ denotes the magnitude of vector $q_{r}$. Then, we utilize $softmax\left(\cdot \right)$ to calculate the normalization attention score. Subsequently, through element-wise product operation, $Z_{r}\left(c\right)$ is weighted to produce an updated version denoted as $Z_{r}^{’}\left(c\right)$. Following this, utilizing $cat\left(\cdot \right)$ and $flatten\left(\cdot \right)$, we redefine the features, resulting in $Z\left(c\right)$. Due to the sharing of filter kernels across nodes within the same scale, we can efficiently extract features from raw EEG data at the corresponding temporal–spatial level. Finally, we obtain the graph node feature encoding $Z^{j}\in R^{C\times SK},\;SK=\sum_{k}^{r}F_{k}^{r}$, for the *j*-th trial of EEG signals.

#### 3.1.2. Task-Specific Adjacency Matrices Construction

Regarding the adjacency matrices, we devised a task-specific construction approach that considers the task or stimulus conditions. This innovative method enables us to incorporate information related to emotional stimuli, thereby enhancing the relevance and applicability of the adjacency matrices to the specific tasks or stimuli under consideration.

Let $X^{j}\in R^{C\times T}$ represent the *j*-th trial of EEG signals, j∈[1,...,N], N means the number of total trials for each subject. The corresponding class label of trial *j* is denoted by $y^{j}$, $y^{j}\in L=\left\{l_{1}=’positive’,l_{2}=’negative’\}\right.$. We can get two clusters of data for the $l_{1}$ and $l_{2}$ ground truth labels, respectively, as follows:

(4) $$X_{l_{1}}=Sel\left(X^{j};y^{j}=l_{1}\right),{\;X}_{l_{2}}=Sel\left(X^{j};y^{j}=l_{2}\right).$$

where the function of $Sel\left(\cdot \right)$ is utilized to select EEG data corresponding to specific emotional stimuli. With the aid of $X_{l_{1}}$ and ${\;X}_{l_{2}}$, we undertake the computation of the adjacency matrix, which serves to represent the connectivity between various EEG channels or nodes within a graph-based brain network. Here, we have opted for four commonly employed functional connectivity methods, namely correlation (Cor), coherence (Coh), phase locking value (PLV), and phase lag index (PLI), all of which have shown promising results in EEG-based emotion recognition [48,49,50,51]. 

Cor provides a direct measure of linear relationship between EEG signals, offering a straightforward assessment of synchrony or covariation, but it is limited in capturing non-linear relationships and is sensitive to outliers and noise. Coh evaluates the consistency of phase differences across frequency bands, providing insights into the synchrony of neural oscillations, yet it may be limited in capturing directional connectivity and can be influenced by volume conduction effects. PLV quantifies phase locking between signals at a specific frequency, being less affected by amplitude fluctuations and focusing solely on phase relationships; however, it does not provide directional information and is sensitive to noise and artifacts. PLI measures the asymmetry in phase differences, useful for identifying directional connectivity between brain regions, yet it ignores zero-phase differences, which may result in the loss of information, and it is also sensitive to noise and artifacts.

As depicted in Equations (5)–(8), for Cor, E, μ and σ  represent expectation, mean, and variance, respectively. The resulting value lies between −1 and 1. A value of 1 indicates that there is a positive correlation between the signals, while a value of −1 indicates negative correlation. A value equal to 0 means that there is no correlation. And for Coh, $S_{x_{a}x_{b}}(f)$ is the cross power spectral density between $x_{a}$ and $x_{b}$, $S_{x_{a}x_{a}}(f)$ and $S_{x_{b}x_{b}}(f)$ are the individual power spectral densities of $x_{a}$ and $x_{b}$. Due to normalization, the value of the coherence lies between 0 and 1. A value close to 1 at a given frequency suggests maximal interdependence between the processes, whereas a value near 0 indicates independence. PLV values range from 0 to 1, with values close to 1 indicating perfect phase locking and values near 0 resulting from random phase distribution. PLI values also range from 0 to 1, with 0 indicating no phase lag and 1 indicating perfect phase lag. Both PLV and PLI are insensitive to signal amplitudes and solely depend on phase relations between the signals.

(5) $${Cor}_{x_{a}x_{b}}=\frac{E\left[\left(x_{a}-\mu_{x_{a}}\right)\left(x_{b}-\mu_{x_{b}}\right)\right]}{\sigma_{x_{a}}\sigma_{x_{b}}}.$$

(6) $${Coh}_{x_{a}x_{b}}=\frac{\left|S_{x_{a}x_{b}}(f)\right|}{\sqrt{S_{x_{a}x_{a}}(f)S_{x_{b}x_{b}}(f)}}.$$

(7) $$PLV=\left|E\left[e^{j∆\phi \left(t\right)}\right]\right|.$$

(8) $$PLI=\left|E\left[sign\left(∆\phi \left(t\right)\right)\right]\right|$$

Each method facilitates the quantification of the functional relationships between EEG channels or nodes, thereby providing valuable insights into the underlying neural dynamics associated with different emotional stimuli. These approaches offer the advantage of capturing variations in the different connectivity patterns of the brain network across different task conditions or emotional states. Each of these measures has its own strengths. For each type of functional connectivity algorithm, we can obtain a pair of task-specific adjacency matrices

(9) $${adj}_{l_{1}}=F\left(X_{l_{1}};\theta \right),\;{adj}_{l_{2}}=F\left(X_{l_{2}};\theta \right);\;\theta \in [Cor,Coh,Plv,Pli]$$

where ${adj}_{l}^{v}\in R^{c\times c},\;v\in \left[Cor,Coh,PLV,PLI\right],l\in \left[l_{1},l_{2}\right]$. In this paper, we separately test each pair of adjacency matrices, and we also employ a parallel integration approach, which will be discussed in Section 5. 

Given the adjacency matrix ${A_{l}^{v}=adj}_{l}^{v}$, the symmetric normalized adjacency matrix can be calculated by

(10) $${\tilde{A}}_{l}^{v}={\tilde{D}}^{-\frac{1}{2}}A_{l}^{v}{\tilde{D}}^{-\frac{1}{2}},$$

where ${\tilde{D}}_{aa}=\sum_{b}A_{l}^{v}\left(a,b\right),and\;a\;and\;b$ are different nodes.

### 3.2. Graph Aggregation with Task-Specific Adjacency Based on Attention

Based on the obtained node feature representations $Z^{j}\in R^{C\times SK}$ and symmetric normalized adjacency matrices $\tilde{A}$, we can proceed to implement GCN learning. For each pair of task-specific adjacency matrices ${\tilde{A}}_{l}^{v}\in R^{c\times c}$, $l\in \left[l_{1},l_{2}\right]$, we will utilize the attention mechanisms to further optimize the aggregation process in GCN, as shown in Figure 2,

(11) $$Z_{l_{1}}^{j}=F_{gcn}\left({\tilde{A}}_{l_{1}},\;Z^{j},W_{l_{1}}\right),\;Z_{l_{2}}^{j}=F_{gcn}\left({\tilde{A}}_{l_{2}},\;Z^{j},W_{l_{2}}\right);Z_{l_{1}}^{j},\;Z_{l_{2}}^{j}\in R^{C\times \left(SK/2\right)}$$

where $F_{gcn}$ represents the GCN learning, and $W_{l_{1}},\;W_{l_{2}}\in R^{SK\times \left(SK/2\right)}$. Subsequently, we advance towards task-specific GCN–attention. We utilize $repeat\left(\cdot \right)$ to construct ${\tilde{Z}}_{l_{1}}^{j},\;{\tilde{Z}}_{l_{2}}^{j}\in R^{C\times C\times SK}$, where each pair of node vectors is concatenated to traverse through, resulting in a matrix of size C×C×SK. Then, employing a pair of trainable projection vectors, denoting $P_{l_{1}}$ and $P_{l_{2}}$, we compute the attention score of ${ascore}_{l_{1}}^{v}$ and ${ascore}_{l_{2}}^{v}$. As shown in Equations (12) and (13),

(12) $${ascore}_{l_{1}}^{v}=F_{att}\left({\tilde{Z}}_{l_{1}}^{j},\;P_{l_{1}}\right),$$

(13) $${ascore}_{l_{2}}^{v}=F_{att}\left({\tilde{Z}}_{l_{2}}^{j},\;P_{l_{2}}\right),$$

(14) $${ascore}_{l_{12}}^{v}={ascore}_{l_{1}}^{v}+{ascore}_{l_{2}}^{v},$$

where $F_{att}$ completes the matrix multiplication of each element, as well as applies non-linear transformation to the attention matrix, ensuring that weighted elements tend towards 1 while unweighted elements become 0. Subsequently, employing $add\left(\cdot \right)$, we obtain ${ascore}_{l_{12}}^{v}$, as shown in Equation (14). Through utilizing ${ascore}_{l_{12}}^{v}$, we modulate ${\tilde{A}}_{l_{1}}$ and ${\tilde{A}}_{l_{2}}$, resulting in ${adjs}_{l_{1}}^{v}$ and ${adjs}_{l_{2}}^{v}$. Consequently, we accomplish the feature aggregation process in GCN, as shown Equation (15),

(15) $$\;{\bar{Z}}_{l_{1}}^{j}=F_{gcn}\left({adjs}_{l_{1}}^{v},\;Z_{l_{1}}^{j},W_{as}\right),\;{\bar{Z}}_{l_{2}}^{j}=F_{gcn}\left({adjs}_{l_{2}}^{v},\;Z_{l_{2}}^{j},W_{as}\right);{\bar{Z}}_{l_{1}}^{j},\;{\bar{Z}}_{l_{2}}^{j}\in R^{C\times \left(SK/4\right)},$$

where $F_{gcn}$ represents the GCN learning, and $W_{as}\in R^{\left(SK/2\right)\times \left(SK/4\right)}$.

### 3.3. Graph Feature Fusion and Classification

**Figure 4:**
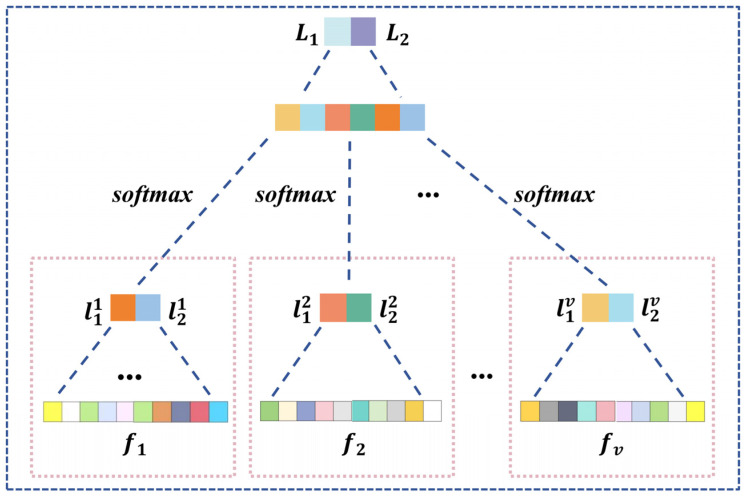
Structure of graph feature fusion and classification.

For different graph aggregation with task-specific adjacency based on attention for $v\in \left[Cor,Coh,PLV,PLI\right]$, we utilize $cat\left(\cdot \right)$ and $flatten\left(\cdot \right)$ to reshape features of ${\bar{Z}}_{l_{1}}^{j,v},\;{\bar{Z}}_{l_{2}}^{j,v}\in R^{C\times \left(SK/4\right)}$, resulting in a set of values denoted as $f_{v}\in R^{C*\left(SK/2\right)}$. Subsequently, we employ multi-layer perceptron function $F_{mlp}^{1}\left(\cdot \right)$ to process $f_{v}$, yielding $l_{1}^{v}$ and $l_{2}^{v}$. By leveraging $softmax\left(\cdot \right)$, $cat\left(\cdot \right)$, and another $F_{mlp}^{2}\left(\cdot \right)$, we implement a weighted two-stage cascaded processing, maximizing the efficacy of task-specific attention, as illustrated in Equation (16) and Figure 4.

(16) $$Z_{out}=F_{mlp}^{2}\left(F_{cat}^{v}\left(F_{softmax}\left(F_{mlp}^{1}\left(f_{v}\right)\right)\right)\right),\;Z_{out}\in \left[\left[L_{1},L_{2}\right]\right].$$

### 3.4. Algorithm for TSANN-TG

In order to optimize the network parameters effectively, we employ the backpropagation technique, iteratively refining them until we reach an optimal or near-optimal solution. To guide this optimization process, we define a loss function based on cross-entropy cost, designed as follows,

(17) $$Loss=cross_{entrony\left(L_{T},L_{P}\right)}+\lambda \left\|\Theta \right\|,$$

where $L_{T}$ and $L_{P}$ represent the true labels and predicted labels of the training datasets, respectively, Θ encompasses all model parameters and λ signifies the trade-off regularization weight. While the cross-entropy function $cross\_entrony\left(L_{T},L_{P}\right)$ quantifies the discrepancy between actual and predicted emotional labels, the regularization term $\lambda \left\|\Theta \right\|$ mitigates over-fitting risks.

Algorithm 1 encapsulates the detailed protocol for training the TSANN-TG model in EEG emotion recognition.
**Algorithm 1:** TSANN-TG   $\mathrm{Input}:\;\mathrm{EEG}\;\mathrm{data}\;X^{j}\;\in \;R^{C\times T}\;$$;\;{label}_{truth}$   **Output:** prediction of the target 1  initialization; 2  **stage1:** 3  temporal 1D CNNs; 4  for r←1 to 3 do 5     for k←1 to 9 do 6        $\;\mathrm{use}\;1D\;\mathrm{CNN}\;\mathrm{temporal}\;\mathrm{kernel}\;\mathrm{with}\;S_{r}^{k}$; 7     **end**
8      kernel attention modulation9  **end** 10  concatenation and flatten calculation 11  calculate task-specific adjacency matrices; 12  **stage2:** 13  $\;\mathrm{first}\;\mathrm{GCN}\;\mathrm{aggregation}\;\mathrm{with}\;W_{l1}$ $\mathrm{and}\;W_{l2\;}$; 14  attention fusion of task-specific adjacency matrices; 15  $\;\mathrm{second}\;\mathrm{GCN}\;\mathrm{aggregation}\;\mathrm{with}\;W_{as}$; 16  graph feature fusion and MLP classifier; 17  **return: prediction**

## 4. Materials and Experiments

In this section, we present the datasets, classification settings and model settings in our experiments.

### 4.1. Emotional EEG Datasets

We validated our proposed TSAN-TG algorithm on two datasets: our own FTEHD (a dataset for face-in-the-crowd task-stimuli emotion recognition of healthy subjects and patients with depression) [52,53], and the publicly available DEAP (a dataset for emotion analysis using EEG, physiological, and video signals) [54].

The FTEHD dataset, approved by the Institutional Review Board of the Shanghai Mental Health Centre (SHMC), comprises data from 16 diagnosed outpatients with depression (Dep) and 14 healthy participants (HC) [52,53]. 

The Dep group were recruited from SMHC and met the diagnosis criteria of major depressive disorder according to the CCMD-3 (the third revision of Chinese Mental Disorder Classification and Diagnosis Standard). Their ages were 37.75 ± 14.19 years old, and they had an average of 12.06 ± 2.91 years of education. None of the HC group had a personal history of neurological or psychiatric illness, and they were not under psychoactive medication. Their ages were 40.86 ± 12.29 years old, and they had an average of 11.54 ± 3.75 years of education. Prior to conducting the experiments, all participants underwent an interview where the Hamilton Rating Scale for Depression (HAMD) was administered. The HAMD scores were 24.5 ± 7.40 for Dep and 7.27 ± 6.94 for HC. Additionally, the participants completed the Self-Rating Anxiety Scale (SAS) and Self-Rating Depression Scale (SDS). The SAS scores were 61.3 ± 9.74 for Dep and 35.5 ± 5.13 for HC, while the SDS scores were 0.89 ± 0.08 for Dep and 0.48 ± 0.09 for HC.

**Figure 5:**
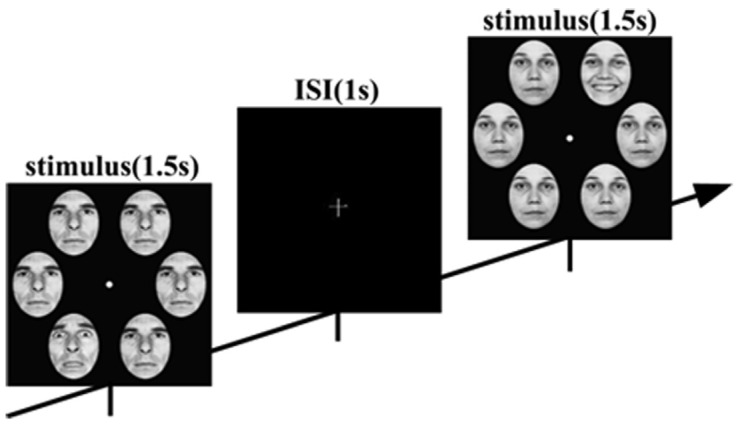
Sequence of trials from the task.

The face-in-the-crowd task stimuli featured six human faces with expressions, selected from the Ekman emotion database, presented in 4 blocks totaling 576 trials each. Participants judged whether the image contained a target face (positive or negative) during a stimulus onset asynchrony, as shown in Figure 5, following a GO-NOGO paradigm. For each trial, segmentations from 200 ms before stimulus onset to 1000 ms post-stimulus were analyzed. EEG signals were recorded at 1000 Hz from 64-channel surface electrodes. Fifty-nine electrodes were selected from 64 electrodes that covered the whole scalp: Fp1, Fpz, Fp2, AF7, AF3, AF4, AF8, F7, F5, F3, F1, Fz, F2, F4, F6, F8, FT7, FC5, FC3, FC1, FC2, FC4, FC6, FT8, T7, C5, C3, C1, Cz, C2, C4, C6, T8, TP7, CP5, CP3, CP1, CPz, CP2, CP4, CP6, TP8, P7, P5, P3, P1, Pz, P2, P4, P6, P8, PO7, PO3, POz, PO4, PO8, O1, Oz, and O2. Data recong was referenced to the tip of the nose. In this paper, the study focuses on classifying EEG signals collected from subjects in the HC group and the DEP group, respectively, as they engage in diverse cognitive tasks, distinguishing between positive and negative stimuli.

The DEAP dataset, a multimodal human affective states dataset comprising EEG, facial expressions, and galvanic skin response, is widely employed in diverse EEG-based emotion recognition research endeavors [54]. Thirty-two subjects (50% female, aged between 19 and 37 with a mean age of 26.9) participated in the data collection experiments, during which EEG signals were captured through a 32-channel device at a sampling rate of 512 Hz. The channels/electrodes used in our study include: Fp1, Fp2, AF3, AF4, F7, F3, Fz, F4, F8, FC5, FC1, FC2, FC6, T7, C3, Cz, C4, T8, CP5, CP1, CP2, CP6, P7, P3, Pz, P4, P8, PO3, PO4, O1, Oz, and O2. Each participant viewed 40 music videos, with each trial lasting 1 min and being preceded by a 3 s baseline. Following each video, participants rated their emotional responses on valence, arousal, dominance, and liking scales ranging from 1 to 9, where 1 represented sad/calm/weak/dislike and 9 indicated happy/excited/strong/like. This paper investigates the classification of EEG signals based on the valence and arousal rating systems.

### 4.2. Experiment Settings

For the FTEHD dataset, we conducted preprocessing to eliminate artifacts, and applied a band–pass filter between 0.05 and 100 Hz. Each trial was labeled as either positive or negative. In this study, we down-sampled the data to a sampling rate of 256 Hz, segmented each trial into 1200 ms windows with an 800 ms window function and 100 ms overlap. Through experimentation, we determined that using window sizes of 800 ms with a 100 ms overlap ensures data validity while effectively capturing its characteristics. This approach expanded the dataset by increasing the number of samples per trial from 1 to 6.

Regarding the DEAP dataset, the 3 s pre-trial baseline was excluded from each trial. Subsequently, the data were down-sampled from 512 Hz to 128 Hz and subjected to a band–pass filter ranging from 4 to 45 Hz. Electrooculogram artifacts were then removed. In our analysis, we utilized the valence and arousal dimension for evaluation purposes. Valence values below 5 were labeled as LV, while those exceeding 5 were labeled as HV. Arousal values below 5 were labeled as LA, while those exceeding 5 were labeled as HA. We employed a 4 s window function with a 2 s overlapping window, which has been proven to be highly effective in multiple experiments. To align with FTEHD expressions, we will also consider HA/HV as positive in some contexts. Similarly, LA/LV corresponds to negative. Therefore, for a 60 s trial, we obtained 29 samples.

Nested cross-validation is a robust technique for evaluating machine learning models [55,56]. It provides unbiased performance estimates by repeatedly splitting the data into training and testing subsets, reducing variance through averaging, aiding in model selection by iteratively testing different configurations, preventing data leakage, and assessing the model’s ability to generalize to unseen data, making it essential for reliable performance evaluation in real-world scenarios.

**Figure 6:**
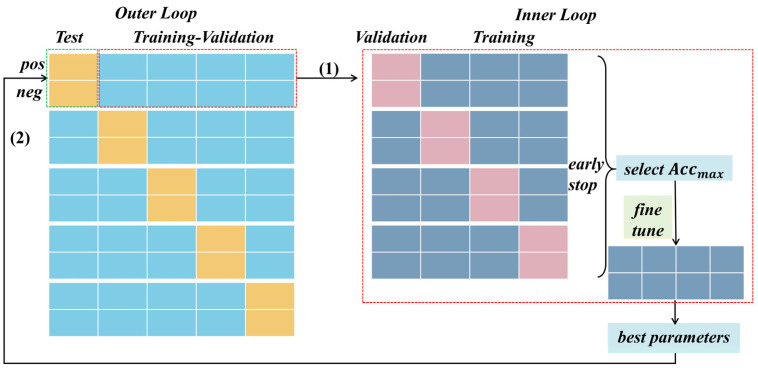
The nested cross-validation based on task stimuli. In both the outer and inner loops, the EEG signals induced by different emotional stimuli were partitioned separately. Within the inner loop, the data slated for testing were excluded, and an early stopping mechanism was employed during validation to refine the models. Subsequently, the best model selected from the four models was subject to fine-tuning using all training–validation data, thereby obtaining optimal parameters for testing on the unseen datasets in the outer loop.

In our study, we implemented nested cross-validation tailored to task stimuli. Illustrated in Figure 6, this methodology comprises an outer loop and an inner loop. Within the outer loop, we employed a 5-fold cross-validation based on positive and negative trials, respectively, for each subject. Specifically, during the testing phase, one-fifth of the positive and negative trial-wise samples were designated as testing data, respectively, while the remaining trials formed the training data. The average accuracy of each cross-validation iteration was then reported as the final evaluation criterion for each dataset.

During the training–validation phase, the inner loop of nested cross-validation involved a 4-fold cross-validation for positive and negative trials. This entailed segregating the training–validation data, excluding the unseen testing data, into folds based on the EEG signals induced by different emotional stimuli (positive and negative). By conducting cross-validation in this manner, we aimed to achieve a more generalized evaluation, considering the distribution of data across different stimuli. Additionally, within each fold’s training process, we integrated an early stopping mechanism, a technique used in machine learning to prevent overfitting. This mechanism continuously monitored the model’s performance on a validation fold throughout training. If the model’s performance failed to improve or began to degrade, training was halted prematurely. This proactive measure prevented the model from excessively fitting the noise present in the training data, thereby enhancing its ability to generalize effectively to unseen data.

Furthermore, the best model from the 4-fold cross-validation was selected, followed by fine-tuning operations on all training–validation data to optimize parameters. These optimized parameters were then utilized to evaluate the performance of each algorithm for each dataset’s test fold, providing the average classification performance.

### 4.3. Performance Assessment

In deep learning, performance assessment is crucial for evaluating the effectiveness of a classifier in classification tasks. Common performance metrics include accuracy (ACC) and F1 measure (F1), derived from the confusion matrix. The confusion matrix compares model predictions with true labels, encompassing True Positive (TP), True Negative (TN), False Positive (FP), and False Negative (FN). It provides a comprehensive overview of classifier performance. TP signifies instances where the classifier accurately predicts the positive class, while TN denotes correct predictions of the negative class. FP arises when the classifier erroneously predicts the positive class in the face of a negative actual class, whereas FN occurs when the classifier incorrectly predicts the negative class amidst a positive actual class.

ACC provides a straightforward measure of overall correctness but may not fully capture performance nuances, particularly in scenarios with imbalanced datasets. Conversely, the F1 score considers both precision and recall, offering a more balanced assessment, crucial in EEG-based emotion recognition where false positives and false negatives carry different implications. However, it is crucial to acknowledge the potential limitations or trade-offs associated with these metrics, such as ACC’s sensitivity to class imbalance and the F1 score favoring either Precision or Recall, depending on the context. A discussion on both indicators would enrich the interpretation of performance evaluation results and provide insights into the reliability of the model’s performance in real-world applications. The calculation formulas for ACC, F1, Precision, and Recall are given by Equations (18)–(20), respectively. Precision measures the accuracy of positive predictions by the model, computed as the ratio of true positives to the sum of true positives and false positives. Conversely, recall, also known as sensitivity or true positive rate, quantifies the proportion of the actual positives correctly identified by the model, calculated as the ratio of true positives to the sum of true positives and false negatives.

(18) $$ACC=\frac{TP+TN}{TP+FP+TN+FN},$$

(19) $$Precision=\frac{TP}{TP+FP},Recall=\frac{TP}{TP+FN},$$

(20) $$F1=\frac{2\times Precision\times Recall}{Precision+Recall},$$

In statistical analysis, selecting the appropriate hypothesis testing method is crucial to ensure the reliability of research findings. In our analysis, we utilized the Wilcoxon signed-rank test at a significance level of α=0.05 to determine the statistical significance of the classification performance observed in our experimental results. This non-parametric test is particularly suitable for comparing paired samples, making it ideal for assessing differences in performance between two related classifiers or algorithms. The Wilcoxon signed-rank test enables us to determine whether any observed disparities are statistically significant, providing valuable insights into the effectiveness of our experimental approaches.

## 5. Results

In this section, we present a comprehensive performance evaluation of our proposed TSANN-TG method. Furthermore, we conduct a comparative analysis against state-of-the-art approaches, categorizing them into two main groups: pure CNN-based models and variants combining CNNs with Graph Neural Networks (CNN-GNN-based models).

The pure CNN-based models considered in our comparison are classical EEGNet [26], DeepConvNet [13,14], ShallowConvNet [13,14], and TSception [28]. On the other hand, the variants combining CNNs with GNNs include 1D CNN-GCN-MLP, 1D CNN-GAT-MLP, and 1D CNN-DGCNN. The approaches of GCN-MLP (graph convolutional network and multi-layer perceptron), GAT-MLP (graph attention network and multi-layer perceptron), DGCNNs (dynamical graph convolutional neural networks) are based on the classical architectures proposed in [32,33,44]. Here, the 1D CNN approach primarily serves for feature extraction, thereby enhancing the performance of the GNN-based models. Notably, the 1D CNN component of these models employs the same scale and quantity of convolutional kernels as our TSANN-TG model.

Additionally, we incorporate SVM-DE and DBN-DE, two methods known for their superior performance in emotion recognition using differential entropy [11,12], as reference models for a comprehensive evaluation. To ensure fairness in comparisons, all baseline methods are fine-tuned with optimal parameters. The experimental results are reported in terms of mean accuracy (mACC) and mean F1 score (mF1) of all subjects, measured on the FTEHD and DEAP datasets, respectively. It is worth noting that all methods are evaluated under standardized settings, and any differences in significance are assessed using the Wilcoxon signed-rank test at a significance level of α=0.05. Moreover, we delve into ablation studies to dissect the components of TSANN-TG, elucidating their individual contributions to overall effectiveness. 

### 5.1. Statistical Analysis

We first report the mACC and mF1 on the two benchmark datasets for four types of cognitive tasks, as shown in Table 1 (for HC group and Dep group) and Table 2 (on valence and arousal).

On the FTEHD dataset, deep learning methods generally outperform non-deep learning methods. Specifically, within the same algorithm, their classification performance in the HC group tends to be superior to that in the Dep group. This observation may stem from the fact that, relative to depressed patients, individuals in the non-depressed group are more likely to elicit emotional responses, leading to more distinguishable EEG signals.

Among the pure CNN-based models, TSception exhibits the highest classification accuracy compared to other methods (89.54%, 89.42%, 84.64%, and 84.36%). Regarding CNN-GNN-based models, 1D CNN-GAT-MLP outperforms the other two methods in terms of mACC for the HC group and the mF1 score for the Dep group (89.69% and 85.12%, respectively), while 1D CNN-DGCNN outperforms the other two methods in terms of the mF1 score for the HC group and mACC for the Dep group (89.39% and 84.98%, respectively). Overall, the best classification performance of CNN-GNN-based models generally surpasses that of CNN-based models, except for the mF1 score for the HC group (89.39% < 89.42%). Classical algorithms such as EEGNet, DeepConvNet, ShallowConvNet, and TSception have all shown significant improvements. Additionally, the combination of CNNs and GNNs tends to yield slightly better results compared to individual models. This may be attributed to the synergistic effect between 1D CNNs in feature extraction and subsequent reinforcement by GNNs, enabling the extraction of more discriminative spatial–temporal features, thus resulting in superior performance.

Additionally, we present the results of our proposed model’s baseline versions, which utilize a single task-specific adjacency, and their performances are superior to all the algorithms above. Notably, TSANN-TG-cor achieves the best performance in mACC for the HC group and the mF1 score for the Dep group (92.1% and 87.25%, respectively), TSANN-TG-plv achieves the best performance in mACC for the Dep group (86.98%), and TSANN-TG-pli achieves the best performance in the mF1 score for the HC group (91.85%). Compared to pure CNN-GNN-based models, we observe further improvement in our model by integrating pairs of task-specific adjacency matrices. This enhancement could be attributed to our approach of conducting graph aggregation with task-specific adjacency based on attention, which renders spatial–temporal features more targeted and endowed with more excellent discriminative properties.

Furthermore, all these performances are lower than those of TSANN-TG fusion (92.28%, 92.62%, 87.39%, and 87.86%, respectively). TSANN-TG fusion significantly outperforms SVM-DE (*p* < 0.001), DBN-DE (*p* < 0.001), EEGNet (*p* < 0.05), DeepConvNet (*p* < 0.05), ShallowConvNet (*p* < 0.05), and 1D CNN-GCN-MLP (*p* < 0.05 for HC). The TSANN-TG fusion model effectively combines the strengths and weaknesses of Cor, COH, PLV, and PLI mentioned earlier in terms of functional connectivity, thereby integrating excellent features from multiple methods and achieving a commendable final outcome.

On the DEAP dataset, similar to the FTEHD dataset, deep learning methods generally outperform non-deep learning methods. Among the pure CNN-based models, DeepConvNet achieves the best performance in the mF1 score on valence (62.23%), while ShallowConvNet achieves the highest accuracy in mACC on valence (60.38%), and TSception attains the best results in mACC on arousal and the mF1 score on arousal (62.78% and 63.62%, respectively). Regarding the CNN-GNN-based models, 1D CNN-GAT-MLP outperforms the other two methods in terms of mACC on valence, and mACC on arousal (61.81% and 62.58%, respectively), and 1D CNN-DGCNN achieves the best performance in the mF1 score on valence and the mF1 score on arousal (62.17% and 63.17%, respectively).

Unlike the results on the FTEHD dataset, we find that the best classification performance of CNN-GNN-based models generally falls below that of CNN-based models, except for the mACC on valence (61.81% > 60.38%).

Similarly, we present the results of our proposed model’s baseline versions, which utilize a single task-specific adjacency, and their performances are superior to all the aforementioned algorithms. Notably, TSANN-TG-cor achieves the best performance in mACC on Arousal and mF1 score on arousal (64.58% and 65.56%, respectively), TSANN-TG-plv achieves the best performance in the mF1 score on valence (64.53%), and TSANN-TG-pli achieves the best performance in mACC on valence (63.15%).

Also, all these performances are lower than those of TSANN-TG fusion (63.74%, 65.28%, 65.93%, and 66.90%, respectively). TSANN-TG fusion significantly outperforms SVM-DE (*p* < 0.01), DBN-DE (*p* < 0.01), EEGNet (*p* < 0.01), DeepConvNet (*p* < 0.05 for mACC on valence and arousal), ShallowConvNet (*p* < 0.05 for mF1 on valence, and *p* < 0.05 for mACC and mF1 on arousal), and 1D CCN-GCN-MLP (*p* < 0.05 for F1 on Valence).

Above, we have conducted an analysis of the valence and arousal tasks in the DEAP dataset. It is evident that the majority of algorithms, particularly those proposed by us, exhibit similar performance to the FTEHD dataset, which strongly corroborates three key observations. Firstly, in our experiments, deep learning methods consistently outperform non-deep learning methods. Secondly, the fusion of CNN-based and GNN-based models effectively extracts superior spatial–temporal features. Thirdly, strategically integrating classical functional connectivity techniques, such as conducting graph aggregation with task-specific adjacency based on attention, fortifies the backbone’s advantages. Moreover, the organic fusion of various functional connectivity methods with distinct strengths can further enhance the model’s performance.

**Figure 7:**
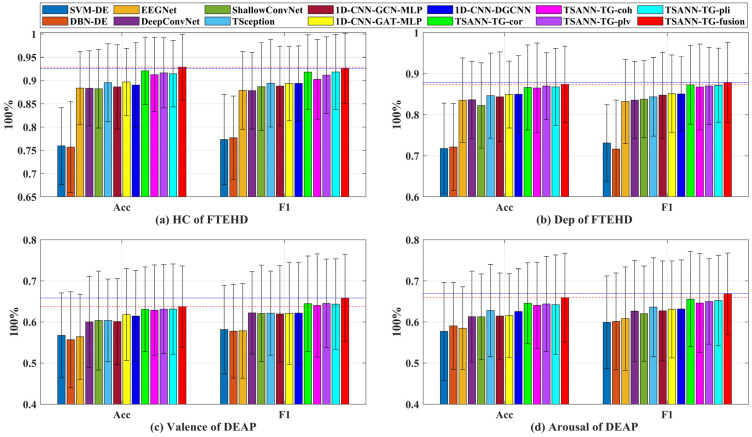
Classification performance of all methods across the two benchmark datasets for four types of cognitive tasks. The red dashed and blue dotted lines in the graph represent the best mACC and mF1 score values for each task, respectively.

Additionally, as depicted in Figure 7, we present the average performance, with bar charts illustrating the classification performance of all methods across the two benchmark datasets for four types of cognitive tasks. The red dashed and blue dotted lines in the graph represent the best mACC and mF1 score values for each task, respectively. It is evident that TSANN-TG fusion consistently achieves the highest recognition rates for each respective task.

Furthermore, we have observed significant discrepancies in the classification performance of the same algorithms between the FTEHD and DEAP datasets. In the FTEHD dataset, each participant’s EEG signals are induced by images, which prompts a more targeted response, facilitating clearer differentiation and resulting in better performance, as demonstrated in this study. In contrast, in DEAP, EEG signals are elicited through video stimuli. Research by Zhang et al. suggests that emotional states fluctuate and manifest discretely over an extended period [57]. From this perspective, trial-wise classification in DEAP is inherently more challenging compared to segment-wise classification, which often yields superior recognition results. As for the trial-wise classification results on DEAP, Ding et al. proposed the state-of-the-art mACC and mF1 scores for valence (59.14% and 62.33%, respectively) and arousal (61.57% and 63.24%, respectively), which are comparable to the performance of TSception, as shown in Table 2 of this paper [28].

**Figure 8:**
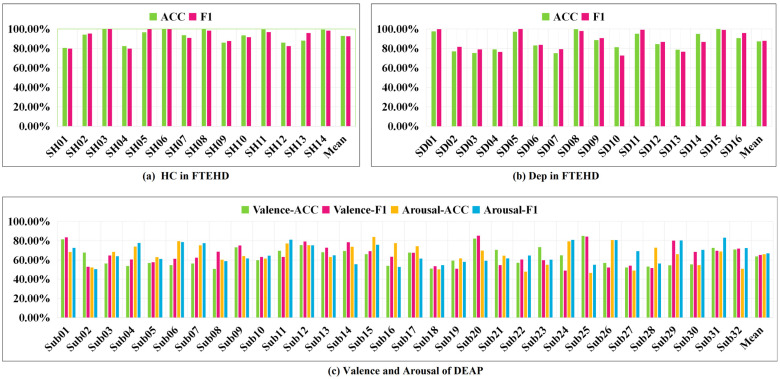
Classification performance of TSANN-TG fusion for each subject.

Additionally, we present the classification performance of TSANN-TG fusion in both the FTEHD and DEAP datasets for each subject, as depicted in Figure 8. Notably, we observe significant discrepancies in cross-validation results among individual subjects, with some exceeding 80% and others falling below 50%. Analyzing the DEAP data provided by Koelstra et al., we found substantial differences in valence–arousal evaluations, even for the same stimulus across different subjects [54]. These findings pose significant limitations on the overall model classification performance, which we will explore further in future research.

### 5.2. Ablation Study

In order to gain a deeper insight into the contributions of each component within TSANN-TG, we conducted ablation experiments by selectively removing the 1D CNN–attention block and the task-specific GCN–attention block. In the first ablation study, the original EEG data were directly fed into the task-specific GCN–attention block. In the second ablation experiment, the feature maps derived from the 1D CNN–attention block were directly flattened and passed into the MLP, resembling the structure of the conventional GCN network. However, it is noteworthy that the operation preserving task-specific adjacency was retained in this modified framework. The ablation here is based on TSANN-TG fusion. A separate section will be set up to explain the effects of different task-specific adjacency.

As demonstrated in Table 3 and Table 4, the removal of either module resulted in a significant decrease in both the mACC and mF1 score. Specifically, for the FTEHD dataset, when considering the HC group, the mACC decreased by over 13.16%, and the mF1 score decreased by over 13.28%. Similarly, for the Dep group, the mACC decreased by over 10.62%, and the mF1 score decreased by over 10.41%. Concerning the DEAP dataset, the ACC for valence decreased by over 7.45%, and the mF1 score decreased by over 7.67%. Likewise, for arousal, the mACC decreased by over 7.63%, and the mF1 score decreased by over 7.57%. 

It is well understood that the removal of either the 1D CNN–attention block or the task-specific GCN–attention block leads to significant alterations in our model’s backbone. In our study, removing the 1D CNN–attention block module essentially means the inability to extract effective features on each channel/electrode/node solely relying on the task-specific GCN–attention block network to aggregate node data. Although distinguishable features can still be obtained, the effectiveness is limited. On the other hand, removing the task-specific GCN–attention block results in limited capabilities in both the temporal and spatial domains, despite the potential to extract some features using the 1D CNN–attention block. Notably, employing the task-specific GCN–attention block alone yields slightly better results than using the 1D CNN–attention block alone. This may be attributed to the effective aggregation of node data by the task-specific GCN–attention block, leading to overall superior performance in both spatial and temporal domains. These findings unequivocally emphasize the effectiveness of both the 1D CNN–attention block and the task-specific GCN–attention block. 

Furthermore, we conducted ablation experiments on the attention mechanisms, as both the temporal and adjacency attention modules were incorporated into the preceding blocks, albeit with slight variations in their implementations. Specifically, we systematically removed one attention module (temporal or adjacency attention) from each block. 

As demonstrated in Table 5 and Table 6. For the FTEHD dataset, upon removing either module individually, we observed a decrease in classification performance of approximately 1% for the HC group and approximately 2% for the Dep group. Notably, when both attention modules were removed simultaneously, the classification performance saw a substantial decline, reaching over 3%. Similarly, for the DEAP dataset, the impact of removing either attention module individually was relatively more intense, with differences exceeding 3% in most cases, except for the mACC and mF1 score on valence (−2.49%, 02.35%, and −2.04%). Furthermore, upon removing both attention modules, the classification performance experienced a decrease of over 4% on arousal. 

It is essential to note that removing either attention module does not alter the backbone of our model. Introducing the temporal attention module in the front part of our model’s backbone enhances the specificity of feature extraction, offering more possibilities for subsequent feature fusion. Additionally, incorporating the adjacency attention into the mid-to-late sections of our model’s backbone facilitates effective feature integration. From a data-driven perspective, the overall effects of incorporating either of the two attention modules are similar, likely due to our model’s consideration of global effects during training. These findings underscore the pivotal roles played by both attention modules in the classification task. 

**Figure 9:**
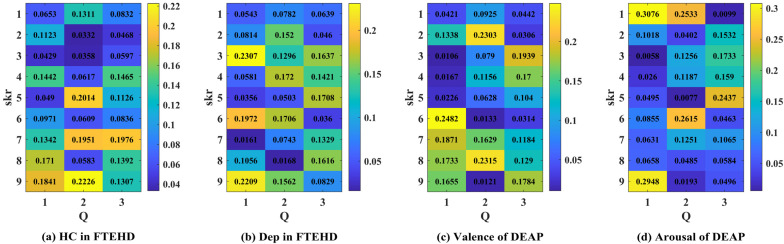
The attention scores of the 1D CNN–attention of TSANN-TG fusion across the two benchmark datasets for four types of cognitive tasks. Note: (**a**–**d**), respectively, denote the results for the HC group in FTEHD, the Dep group in FTEHD, the valence of DEAP, and the arousal of DEAP.

**Figure 10:**
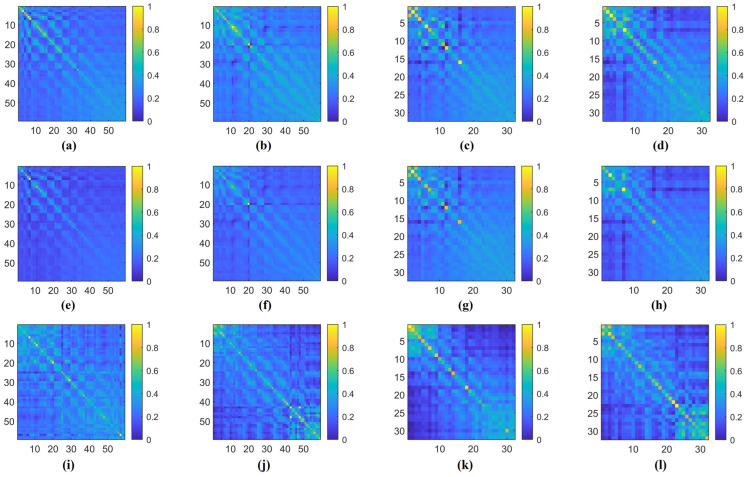
The task-specific adjacency matrices and GCN–attention scores of TSANN-TG fusion across the two benchmark datasets for four types of cognitive tasks. Notably, (**a**,**e**,**i**) represent results for the HC group in FTEHD, while (**b**,**f**,**j**) denote those for the Dep group in FTEHD. Similarly, (**c**,**g**,**k**) correspond to results for the valence of DEAP, and (**d**,**h**,**l**) denote those for the arousal of DEAP.

Additionally, in Figure 9, we illustrate an example of the 1D CNN–attention process on the two benchmark datasets for four types of cognitive tasks. Observing the respective Q values in each type of cognitive task, we notice variations in the weight magnitudes of Q1, Q2, and Q3 across nine different levels of filter kernels. However, each one exhibits prominent components. Leveraging gradient descent backpropagation algorithms, this final aggregation facilitates swift optimization across multiple dimensions. In Figure 10, we present the task-specific adjacency matrices and GCN–attention scores of TSANN-TG fusion across the two benchmark datasets for four types of cognitive tasks. For the FTEHD dataset, the first and second rows, respectively, denote the positive and negative components in the task-specific adjacency matrices, while the third row represents GCN–attention scores. Similarly, for the DEAP dataset, the first and second rows represent HV/HA and LA/LV components in the task-specific adjacency matrices, and the third row indicates GCN–attention scores. Notably, compared to the first and second rows, the connectivity patterns in the third row exhibit greater global coherence, potentially empowering more robust feature map learning.

### 5.3. Effects of Fusion Task-Specific GCN–Attention Block

Furthermore, to better understand our multitask-specific GCN–attention block fusion mechanism, we conducted comparative experimental analyses on different combinations of tAdj-cor, tAdj-coh, tAdj-plv, and tAdj-pli.

As illustrated in Table 7 and Table 8, we observed that, for the FTEHD dataset, in general, the fusion of three groups yielded superior results compared to the fusion of two groups. However, intriguingly, we found that in the experiments for mF1 scores on HC and Dep, the best classification performance achieved by the fusion of two groups surpassed that of the best performance achieved by the fusion of three groups (92.59% vs. 92.54%, and 87.85% vs. 87.82%, respectively), while remaining remarkably close to the performance obtained by the fusion of four groups. Regarding the DEAP dataset, a similar trend was observed, where, overall, the fusion of three groups yielded slightly better results compared to the fusion of two groups. However, notably, in the mF1 scores on valence and mACC on arousal, the best classification performance achieved by the fusion of two groups outperformed that of the best performance achieved by the fusion of three groups (65.59% vs. 65.58%, and 65.86% vs. 65.82%, respectively). 

The above findings show that integrating various functional connection matrices enhances the network’s adaptability from diverse perspectives, allowing it to encompass as much pertinent information as possible during training. The four classical functional connection methods chosen in this study aptly evaluate the interrelation between EEG signals in terms of time domain, frequency domain, and phase, providing strong assurance for achieving good results.

## 6. Discussion

Our comprehensive evaluation underscores significant advancements in EEG-based emotion recognition facilitated by TSANN-TG. Across various cognitive tasks and datasets, we observe remarkable enhancements in both mACC and mF1 scores. Specifically, TSANN-TG surpasses traditional methods, as well as existing CNN-based and CNN-GNN-based models on both the FTEHD and DEAP datasets. These improvements range from 3.19% to 17.18% in mACC for the HC group on the FTEHD dataset, from 3.23% to 15.29% in mF1 for the HC group on the FTEHD dataset, from 2.41% to 15.62% in mACC for the Dep group on the FTEHD dataset, from 2.74% to 16.2% in mF1 for the Dep group on the FTEHD dataset, from 1.93% to 8.04% in mACC for valence on the DEAP dataset, from 3.11% to 7.51% in mF1 for valence on the DEAP dataset, from 3.35% to 8.2% in mACC for arousal on the DEAP dataset, and from 3.73% to 6.98% in mF1 for arousal on the DEAP dataset. These findings underscore the efficacy of TSANN-TG in pushing the boundaries of EEG-based emotion recognition methodologies. 

In our comparative experiments, we have selected a set of exemplary algorithms including SVM-DE, DBN-DE, EEGNet, DeepConvNet, ShallowConvNet, and TSception. To optimize the performance assessment of GNN-based algorithms, we have taken a deliberate approach of furnishing GNN networks (specifically, GCN, GAT, and DGCNN) with refined features rather than raw data. This strategy ensures a robust and dependable comparative analysis, enhancing our ability to discern the strengths and nuances of each algorithm under scrutiny. The results demonstrate that our method outperforms current state-of-the-art methods, whether traditional, CNN-based, or GNN-based. This superiority may stem from our method’s incorporation of distinct attention mechanisms during feature extraction and fusion stages, coupled with a task-specific graph strategy, which comprehensively mines effective information, ultimately yielding impressive results.

In our ablation study, we systematically dissected the contributions of each component within TSANN-TG by selectively removing the 1D CNN–attention block or the task-specific GCN–attention block. The results revealed significant decreases in classification performance upon the removal of either module, emphasizing their critical roles in enhancing feature extraction and capturing dynamic dependencies between EEG channels. 

The 1D CNN–attention block plays a pivotal role in extracting frequency-specific information. By leveraging the attention mechanisms, this module enables targeted extraction of relevant features, allowing us to identify key information within EEG signals. Additionally, the shared parameters of filter kernels and trainable projection vectors across nodes enhance the efficiency of feature extraction and facilitate the construction of node feature representations for the graph network. 

On the other hand, the task-specific GCN–attention block contributes to the improved aggregation of node features in the original GCN. This enhanced aggregation mechanism maximizes the utilization of task-specific and spatially connected information, further improving the model’s performance in emotion recognition tasks.

We investigate the effects of different combinations of task-specific adjacency matrices on classification performance. The results indicate that the fusion of multiple task-specific adjacency matrices generally yields superior results compared to individual matrices. However, intriguingly, we observe instances where the fusion of two groups outperforms the fusion of three groups, suggesting the importance of selecting an optimal combination of adjacency matrices tailored to specific cognitive tasks.

Building upon the task–stimuli paradigms, the nested cross-validation methodology intricately harnesses the wealth of information present in training–validation data while vigilantly guarding against data leakage pitfalls. This approach not only fortifies the robustness of our model but also enhances its generalization efficacy by providing a stringent validation framework. Through this methodology, we achieve a more accurate appraisal of performance metrics, ensuring that our TSANN-TG model can effectively generalize to unseen data while maintaining reliable performance across diverse tasks and stimuli paradigms.

Overall, our study underscores TSANN-TG’s efficacy and versatility in EEG-based emotion recognition. The targeted extraction mechanism is particularly beneficial for enhancing the effectiveness of feature representations in graph networks. By incorporating task-specific attention mechanisms, it enables more effective integration of spatial information and task-specific functionality within the network. The findings provide valuable insights into effective feature extraction and classification mechanisms, contributing to advancements in this field.

TSANN-TG’s groundbreaking advancements in EEG-based emotion recognition not only drive the field forward but also hold immense promise for practical applications. Its exceptional accuracy and robustness pave the way for the development of more intuitive affective BCIs and significantly enhance mental health assessments. By offering reliable monitoring and timely interventions for mood disorders, TSANN-TG plays a pivotal role in improving emotional well-being in real-world settings. Additionally, acknowledging the limitations of our study, such as individual differences in EEG patterns and the need for further validation on real-world emotion recognition tasks, will be crucial for guiding future research endeavors and ensuring the continued advancement of EEG-based emotion recognition technology. 

## 7. Conclusions

Our work introduces TSANN-TG, a novel CNN-GNN model architecture inspired by neurology, designed to comprehend brain activities across various temporal and spatial regions. TSANN-TG comprises the following three primary components: (a) a node-feature-encoding-and-adjacency-matrices-construction block, specialized in capturing frequency-specific features based on an attention mechanism and generating task-specific adjacency matrices; (b) a graph aggregation block, dedicated to integrating task-specific information into the graph structure using an attention mechanism; and (c) a graph-feature-fusion-and-classification block, responsible for fusing features from diverse graph representations and executing the final classification. A comprehensive evaluation of both datasets showcased TSANN-TG’s superior performance in terms of accuracy and F1 score across the four types of cognitive tasks, outperforming other baseline algorithms on these two benchmark datasets. 

In conclusion, our proposed TSANN-TG architecture presents a significant advancement in EEG-based emotion recognition for aBCIs. Future research can further explore and refine TSANN-TG to achieve even greater performance in emotion recognition tasks, ultimately benefiting applications in affective brain–computer interfaces and related fields. Furthermore, future studies could explore integrating TSANN-TG with other modalities, such as facial expressions or physiological signals, and investigate its performance on larger and more diverse datasets, thus enhancing its applicability and effectiveness in real-world scenarios. 

## Figures and Tables

**Table 1:** Classification performance of different methods for FTEHD datasets.

Method	HC Group				Dep Group			
	mACC	std	mF1	std	mACC	std	mF1	std
SVM-DE	75.95% (***)	8.23%	77.33% (***)	9.71%	71.77% (***)	11.06%	73.12% (***)	9.32%
DBN-DE	75.70% (***)	9.76%	77.70% (***)	8.97%	72.15% (***)	10.59%	71.66% (***)	11.93%
EEGNet	88.38% (*)	7.87%	87.88% (*)	8.36%	83.53% (*)	10.31%	83.23% (*)	10.26%
DeepConvNet	88.33% (*)	8.05%	87.83% (*)	8.24%	83.64% (*)	9.39%	83.56% (*)	9.41%
ShallowConvNet	88.24% (*)	8.45%	88.70% (*)	9.40%	82.25% (*)	10.40%	83.79% (*)	9.40%
TSception	89.54%	8.39%	89.42%	9.41%	84.64%	10.35%	84.36%	9.58%
1D CNN-GCN-MLP	88.64% (*)	9.05%	88.82% (*)	8.57%	84.35%	10.94%	84.76%	10.47%
1D CNN-GAT-MLP	89.69%	7.23%	89.38%	8.02%	84.92%	8.15%	85.12%	9.42%
1D CNN-DGCNN	89.02%	9.13%	89.39%	8.06%	84.98%	9.38%	85.07%	9.03%
TSANN-TG-cor	92.10%	7.23%	91.82%	8.02%	86.64%	10.35%	87.25%	9.58%
TSANN-TG-coh	91.27%	7.95%	90.26%	8.57%	86.53%	10.94%	86.76%	10.47%
TSANN-TG-plv	91.66%	7.53%	91.16%	8.24%	86.98%	8.15%	87.02%	9.42%
TSANN-TG-pli	91.46%	7.13%	91.85%	8.06%	86.79%	9.38%	87.17%	9.03%
TSANN-TG fusion	92.88%	7.09%	92.62%	7.57%	87.39%	9.32%	87.86%	9.78%

*p*-value between the TSANN-TG fusion and other methods: * means (*p* < 0.05) and *** means (*p* < 0.001).

**Table 2:** Classification performance of different methods for DEAP datasets.

Method	Valence				Arousal			
	mACC	std	mF1	std	mACC	std	mF1	std
SVM-DE	56.75% (**)	10.32%	58.17% (**)	10.79%	57.73% (**)	11.90%	59.92% (**)	11.29%
DBN-DE	55.70% (**)	11.67%	57.77% (**)	11.38%	59.05% (**)	10.59%	60.16% (**)	11.78%
EEGNet	56.41% (**)	10.34%	57.87% (**)	11.50%	58.49% (**)	10.08%	60.83% (**)	12.56%
DeepConvNet	60.02% (*)	11.07%	62.23%	10.02%	61.32% (*)	11.05%	62.64%	12.32%
ShallowConvNet	60.38%	12.03%	62.08% (*)	11.76%	61.26% (*)	10.45%	62.03% (*)	11.58%
TSception	60.36%	10.08%	62.16%	10.25%	62.78%	11.23%	63.62%	12.03%
1D CNN-GCN-MLP	60.09%	10.49%	61.97% (*)	11.74%	61.45%	10.49%	62.71%	12.17%
1D CNN-GAT-MLP	61.81%	11.18%	62.09%	12.47%	62.58%	10.18%	63.08%	11.79%
1D CNN-DGCNN	61.45%	11.13%	62.17%	12.37%	62.56%	10.39%	63.17%	11.96%
TSANN-TG-cor	63.08%	10.30%	64.46%	11.63%	64.58%	9.86%	65.56%	11.60%
TSANN-TG-coh	62.86%	11.02%	64.03%	12.56%	64.05%	10.50%	64.63%	12.06%
TSANN-TG-plv	63.12%	10.83%	64.53%	10.78%	64.41%	11.60%	65.00%	10.42%
TSANN-TG-pli	63.15%	10.98%	64.36%	11.03%	64.23%	12.10%	65.23%	11.03%
TSANN-TG fusion	63.74%	9.90%	65.28%	10.59%	65.93%	10.80%	66.90%	9.88%

*p*-value between the TSANN-TG fusion and other methods: * means (*p* < 0.05) and ** means (*p* < 0.01).

**Table 3:** Ablation performance of the 1D CNN–attention block and the task-specific GCN–attention block for the FTEHD dataset.

Method		HC				Dep			
1D CNN–Attention	Task-Specific GCN–Attention	mACC	Variation	mF1	Variation	mACC	Variation	mF1	Variation
	√	79.72%	−13.16%	79.34%	−13.28%	76.77%	−10.62%	77.45%	−10.41%
√		77.86%	−15.02%	79.15%	−13.47%	75.09%	−12.30%	76.17%	−11.69%
√	√	92.88%	-	92.62%	-	87.39%	-	87.86%	-

√: keep this component.

**Table 4:** Ablation performance of the 1D CNN–attention block and the task-specific GCN–attention block for the DEAP dataset.

Method		Valence				Arousal			
1D CNN–Attention	Task-Specific GCN–Attention	mACC	Variation	mF1	Variation	mACC	Variation	mF1	Variation
	√	56.29%	−7.45%	56.45%	−8.83%	58.30%	−7.63%	59.33%	−7.57%
√		56.20%	−7.54%	57.61%	−7.67%	58.26%	−7.67%	59.17%	−7.73%
√	√	63.74%	-	65.28%	-	65.93%	-	66.90%	-

√: keep this component.

**Table 5:** Ablation performance of the attention mechanisms for FTEHD dataset.

Method		HC				Dep			
Temporal Attention	Adjacency Attention	mACC	Variation	mF1	Variation	mACC	Variation	mF1	Variation
		88.98%	−3.90%	89.36%	−3.26%	84.36%	−3.03%	84.38%	−3.48%
√		91.02%	−1.86%	90.26%	−0.72%	85.43%	−1.96%	85.78%	−2.08%
	√	91.33%	−1.55%	90.98%	−1.64%	85.59%	−1.80%	85.36%	−2.50%
√	√	92.88%	-	92.62%	-	87.39%	-	87.86%	-

√: keep this component.

**Table 6:** Ablation performance of the attention mechanisms for DEAP dataset.

Method		Valence				Arousal			
Temporal Attention	Adjacency Attention	mACC	Variation	mF1	Variation	mACC	Variation	mF1	Variation
		60.07%	−3.67%	61.99%	−3.29%	61.76%	−4.17%	62.89%	−4.01%
√		61.25%	−2.49%	62.17%	−3.11%	62.49%	−3.44%	63.43%	−3.47%
	√	61.39%	−2.35%	63.24%	−2.04%	62.54%	−3.39%	63.26%	−3.64%
√	√	63.74%	-	65.28%	-	65.93%	-	66.90%	-

√: keep this component.

**Table 7:** Classification performance of different combinations with tAdj-cor, tAdj-coh, tAdj-plv, and tAdj-pli for FTEHD dataset.

Method				HC				Dep			
tAdj-cor	tAdj-coh	tAdj-plv	tAdj-pli	mACC	Variation	mF1	Variation	mACC	Variation	mF1	Variation
√	√			92.11%	−0.77%	91.84%	−0.78%	86.76%	−0.63%	87.85%	−0.01%
√		√		92.18%	−0.71%	92.59%	−0.03%	86.95%	−0.44%	87.35%	−0.51%
√			√	92.13%	−0.75%	91.85%	−0.77%	87.27%	−0.12%	87.28%	−0.58%
	√	√		91.87%	−1.02%	91.21%	−1.41%	86.43%	−0.96%	86.96%	−0.90%
	√		√	91.71%	−1.17%	91.87%	−0.75%	86.35%	−1.04%	87.18%	−0.68%
		√	√	91.82%	−1.06%	91.83%	−0.79%	86.46%	−0.93%	87.29%	−0.57%
√	√	√		92.15%	−0.74%	91.97%	−0.65%	87.08%	−0.31%	87.82%	−0.04%
√	√		√	92.65%	−0.23%	92.21%	−0.41%	86.93%	−0.46%	87.62%	−0.24%
√		√	√	92.19%	−0.69%	92.54%	−0.08%	87.34%	−0.05%	87.53%	−0.33%
	√	√	√	92.37%	−0.51%	91.76%	−0.86%	87.28%	−0.11%	87.78%	−0.08%
√	√	√	√	92.88%	-	92.62%	-	87.39%	-	87.86%	-

√: add this component.

**Table 8:** Classification performance of different combinations with tAdj-cor, tAdj-coh, tAdj-plv, and tAdj-pli for DEAP dataset.

Method				Valence				Arousal			
tAdj_cor	tAdj_coh	tAdj_plv	tAdj_pli	mACC	Variation	mF1	Variation	mACC	Variation	mF1	Variation
√	√			63.09%	−0.65%	64.19%	−1.67%	64.25%	−1.68%	65.32%	−1.58%
√		√		63.59%	−0.15%	65.08%	−0.78%	65.46%	−0.47%	65.65%	−1.25%
√			√	63.41%	−0.33%	65.59%	−0.27%	65.86%	−0.07%	65.99%	−0.91%
	√	√		63.29%	−0.45%	64.39%	−1.47%	64.32%	−1.61%	66.09%	−0.81%
	√		√	63.17%	−0.57%	64.56%	−1.30%	64.25%	−1.68%	66.27%	−0.63%
		√	√	63.14%	−0.60%	64.76%	−1.10%	64.66%	−1.27%	66.03%	−0.87%
√	√	√		63.15%	−0.59%	65.29%	−0.57%	64.94%	−0.99%	66.58%	−0.32%
√	√		√	63.30%	−0.44%	65.26%	−0.60%	65.65%	−0.28%	66.83%	−0.07%
√		√	√	63.64%	−0.10%	65.58%	−0.28%	65.82%	−0.11%	66.42%	−0.48%
	√	√	√	63.53%	−0.21%	65.51%	−0.35%	65.75%	−0.18%	66.77%	−0.13%
√	√	√	√	63.74%	-	65.86%	-	65.93%	-	66.90%	-

√: add this component.

## Data Availability

The datasets are available upon request from the corresponding author. The data are not publicly available due to privacy reasons.
